# Investigations on the Effects of Different Calcium Supply Exceeding the Requirements on Mineral Serum Concentrations and Bone Metabolism in Young Warmblood Stallions

**DOI:** 10.3390/ani11082439

**Published:** 2021-08-19

**Authors:** Dana Carina Schubert, Lisa-Theresa Neustädter, Manfred Coenen, Christian Visscher, Josef Kamphues

**Affiliations:** 1 Institute for Animal Nutrition, University of Veterinary Medicine Hannover, Foundation, Bischofsholer Damm 15, D-30173 Hanover, Germany; Christian.Visscher@tiho-hannover.de (C.V.); Josef.Kamphues@tiho-hannover.de (J.K.); 2 Tierärztliche Klinik für Pferde Dres. Marcus Bayer, Wigo Horstmann, Johanna Engl, Breite Straße 141, D-67067 Ludwigshafen, Germany; Lisa.Neustaedter@hotmail.de; 3 Institute of Animal Nutrition, Nutrition Diseases and Dietetics, Faculty of Veterinary Medicine, Leipzig University, An den Tierkliniken 9, D-04103 Leipzig, Germany; Manfredcoenen@web.de

**Keywords:** bulk elements, minerals, horse, equine nutrition, requirement

## Abstract

**Simple Summary:**

In horse husbandry, it is a common practice to supplement roughages with mineral or complementary feeds, which contain, among others, macro elements. However, roughages usually fulfil the requirements for macro minerals but not of trace elements of horses at maintenance. Therefore, in the present feeding trial mineral feeds with different calcium levels moderately exceeding the requirements vs. 2-fold higher levels were tested in young stallions (2–3 years old) for five months under practical conditions. Blood samples of the stallions were analysed to prove whether the feeding regimes influence mineral status or bone metabolism. The results suggest that reduced calcium intake favours phosphorus levels in the blood, but does not affect serum levels of trace elements and bone metabolism in young, still growing stallions.

**Abstract:**

Since mineral supplements for horses commonly contain macro minerals, although the requirement for such is usually covered by roughage-based diets, the aim of this study was to investigate the effects of different dietary calcium levels on mineral serum concentrations and bone metabolism. The trial was conducted in 30 young warmblood stallions (2–3 years) that were divided into two groups for a five-month feeding trial. The groups were fed a hay- and oat-based diet and were either supplied with high (Ca-High) or moderate (Ca-Moderate) calcium excess. While in Ca-High calcium supply was about 2–2.5-fold of the requirement, in Ca-Moderate calcium requirements were slightly surpassed (1.5–1.6-fold). In order to monitor the effects of the different calcium supply, blood samples were taken during the trial and analysed for levels of macro and trace elements as well as concentrations of two bone markers. In Ca-Moderate a trend towards higher phosphorus serum levels compared to Ca-High was observed which was significant at the end of the trial (*p* = 0.0002). Furthermore, results showed no influence of the diet on bone markers. Results support the idea that forage-based rations for horses do not necessarily have to be supplemented with macro minerals but with trace elements.

## 1. Introduction

Mineral supplements for horses (*Equus caballus*) usually include both, macro as well as trace elements. However, forage-based diets, which are common in horse feeding, already contain adequate levels of macro minerals, especially sufficient levels of calcium. According to the recommendation by the Committee for Requirement Standards of the Society of Nutrition Physiology (GfE) from 2014 [1], the calcium requirement of a warmblood horse (bodyweight 600 kg, 25 to 36 months old) is 25.8 g per day. This amount is already covered by about 5.5 kg of hay, with an average calcium content in grass hay of 4.8 g per kg dry matter (variations from 1.6–19.4 g/kg DM were observed in hay samples from the years 2014 to 2019 in Germany) [2]. In other types of hay, for example, clover or alfalfa hay, the calcium contents are even higher than those found in grass hay and reach mean concentrations of 15.7 g/kg DM [3].

Reducing the macro-mineral supply might improve the utilisation of specific minerals, as high levels of calcium in the diet were reported to cause a secondary deficiency of trace elements [4]. In horses, high dietary calcium negatively affected copper digestibility, whereas zinc digestibility was not influenced [5]. In pigs (*Sus scrofa domesticus*), carnivores and small ruminants, on the other hand, a secondary zinc deficiency due to high dietary calcium levels was observed [3]. In addition, reduced serum magnesium concentrations were found in horses with increased calcium and phosphorus levels in the diet [6]. In terms of the physiological calcium metabolism, horses differ from other mammalian species, as a dietary calcium excess is not only excreted with faeces but also via urine [7]. This bears the risk of urinary stones with increased calcium levels in the feed [8].

Basically, the supply level of minerals is reflected in the serum of horses, so blood tests provide a simple yet reliable criterion for assessment [9]. However, the assessment of calcium supply via a blood test is only of limited value, since calcium levels in the blood are strictly regulated by endocrine mechanisms. In terms of insufficient calcium supply, parathyroid hormone (PTH) which is secreted by the parathyroid glands causes the release of calcium from the skeleton by stimulating osteoclastic bone resorption and leads to increased tubular reabsorption of calcium in the kidney. Furthermore, PTH stimulates the activation of vitamin D into its biologically active form calcitriol (1,25-dihydroxycholecalciferol), which in turn causes an increased intestinal calcium absorption [10]. By means of balance studies, an exact determination of the intestinally absorbed calcium amount, as well as the renal calcium excretion, is possible. However, performing balance studies means enormous restrictions on the animals’ welfare. Nevertheless, to investigate the effects of different dietary calcium levels in more detail, bone markers can be used in order to monitor bone metabolism, which might be of interest, especially in growing animals. For this purpose, plasma levels of osteocalcin (OC) can provide information about bone formation as it is a synthesis product of osteoblasts and is produced during matrix formation [11,12]. In contrast, levels of C-terminal telopeptide (CTX-I) can give information about degradation processes [13]. CTX-I fragments are released into the blood as degradation products of type I collagen during osteoclast-mediated bone resorption [14].

The effects of different calcium contents in the diet of warmblood horses in the late growth phase (25- to 36-months-old) have been hardly researched. Our hypothesis is that high calcium excess might impair the utilisation of trace elements. To prove this theory, the effects on serum mineral status and bone metabolism in young stallions fed a hay- and oat-based diet were evaluated under conditions of either high or moderate calcium excess.

## 2. Materials and Methods

The experiments were conducted in accordance with German regulations and approved by the Ethics Committee of Lower Saxony for the Care and Use of Laboratory Animals (LAVES: Niedersächsisches Landesamt für Verbraucherschutz und Lebensmittelsicherheit; reference: 33.9-42502-05-14A473).

### 2.1. Animals and Housing

The study was conducted between November 2014 and April 2015 in initially 32 warmblood stallions born in 2012 (mean age 963 ± 25 d at the beginning of the trial) with an initial body weight of 536 ± 32 kg. The individual age as well as the starting and final weight of the stallions can be found in the supplementary material Table S1. The animals were divided into two groups (Ca-High and Ca-Moderate) according to body weight and pedigree. Two animals from Ca-High were removed from the study, one due to colic and one due to sale, resulting in different numbers of animals in the two groups (Ca-High: *n* = 14, Ca-Moderate: *n* = 16). The groups were kept in two identical stables at a stallion station in Lower Saxony, Germany. Each stable contained 16 boxes of equal size (3.5 × 4.4 = 15.4 m^2^) that were littered with straw and equipped with automatic drinkers. The horses were exercised daily for 30 min (light work).

### 2.2. Feeding Concept and Diets

The trial was split into two phases. The feeding concept during phase 1 (week 0–week 10) was built on supplementing macro and trace elements via a mineral feed (MF; small daily amount of about 100 g necessary) when feeding a hay- and oat-based diet and additional concentrates, soybean meal and oil. However, due to the reduction in calcium, CF_2_ did not meet the definition of a mineral feed (min. 40% ash) according to Regulation (EC) No 767/2009 [15] but was based on the concept of a mineral feed. During phase 2 (week 11–week 20), minerals were supplemented via complementary feeds (CF; daily amount of 1 kg necessary) replacing concentrates and soybean meal, so that horses received only oats and oil in addition to the hay-based diet and the CF. The quantities of the components of the different rations can be taken from Table 1.

The composition of the MF and CF is shown in Table 2. MF_1_ and CF_1_ were designed to create a high calcium excess (Ca-High) whereas MF_2_ and CF_2_ were designed to create a moderate calcium oversupply (Ca-Moderate). The chemical composition of the remaining feedstuffs (hay, oats, concentrates, soybean meal) and the straw used as bedding material can be found in the supplementary material Tables S2 and S3. Hay was analysed weekly, so that the displayed values represent the means of the weekly results, while straw was analysed once in each phase. In terms of MFs, CFs, concentrates and oats, a representative sample was taken from the batch and analysed before the start of the trial. For the soybean meal, the contents given by Coenen and Vervuert [16] were used for the calculations.

For each ration, the total daily intake of nutrients was calculated assuming a dry matter intake of 12 kg per animal achieved by an additional intake of straw from the litter (Table 3 and Table 4). In Table 3, the intake of minerals is compared to the requirements according to the GfE [1] (for an assumed dry matter intake of 12 kg per animal and day).

In Ca-High the calcium supply was 2.5-fold (phase 1) and 1.9-fold (phase 2), respectively, of the requirement, whereas in Ca-Moderate the calcium supply was 1.6-fold (phase 1) and 1.5-fold (phase 2), respectively, of the requirement according to the GfE [1].

The horses were fed three times a day (6:15, 11:30 and 17:00 h, respectively) at equal shares. Feed intake was controlled by the stable staff. No feed refusals were observed, therefore, the feed intake was equated with the feed supply. However, small losses in the bedding material (straw) cannot be excluded. In addition to mineral supply via MF or CF, access to a salt-lick-stone (NaCl) was provided to free access to each animal in both groups.

### 2.3. Experimental Procedure and Sampling

**Figure 1:**
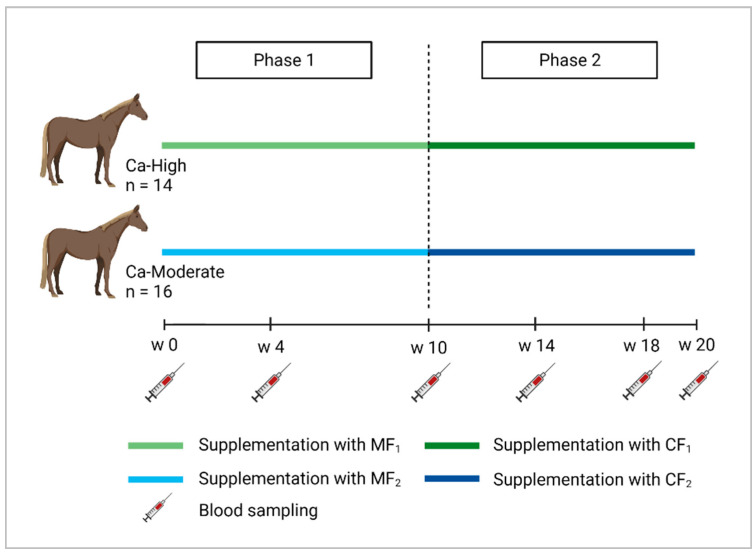
Scheme of the experimental procedure and sampling protocol.

Blood samples were obtained in order to determine concentrations of minerals (Ca, P, Mg, Na, K, Cl, Fe, Cu, Zn, Se) and the bone markers osteocalcin (OC) and C-terminal telopeptide (CTX-I). Blood samples were taken at the beginning of the trial (week 0) and then at week 4, week 10, week 14, week 18, and at the end of the trial (week 20) by puncturing the vena jugularis externa using plasma and serum monovettes (S-Monovette^®^ K3-EDTA, 9 mL, S-Monovette^®^ Serum, 9 mL, Sarstedt AG and Co., Nümbrecht, Germany). The blood samples were always taken at the same time between 13:00 and 14:00 h. Furthermore, bodyweight was recorded five times throughout the trial (week 0, week 5, week 11, week 19, and week 20). Figure 1 shows a scheme of the experimental procedure.

### 2.4. Analytical Methods

Feeding materials were analysed by standard procedures in accordance with the official methods of VDLUFA [17]. To determine dry matter (DM), samples were dried at 103 °C until weight constancy. By incinerating the samples at 600 °C in the muffle furnace, the crude ash was analysed. The total nitrogen content was determined by using a catalytic tube combustion method (DUMAS combustion method; Vario Max^®^, Elementar Analysensysteme GmbH, Langenselbold, Germany). By multiplying total N with a constant factor of 6.25, the crude protein content was calculated. The ether extract was determined after acid hydrolysis in the Soxleth apparatus. The crude fibre was analysed after washing the samples in dilute acids and alkalis (Fibertec 2010 Hot Extraktor^®^, Foss, Hilleroed, Denmark). The NDF was analysed according to Van Soest et al. [18] by using the Fibertec M6 1020 (Foss, Hilleroed, Denmark). NDF was not corrected for crude ash. Mineral contents (Ca, Mg, K, Na, Fe, Zn, Cu, Se) were determined by atomic absorption spectrometry (Solaar M Series Atomic Absorption Spectrometer, Thermo Elemental, Cambridge, England). Colorimetric measurement was applied to detect the phosphorus content (UV-Visible Recording Spectrophotometer UV 162, Shimadzu, Kyoto, Japan), whereas colorimetric titration was used to analyse the chloride content (Chloride Analyzer 925, Ciba Corning Diagnostics, Medfield, MA, USA).

For the blood analysis, plasma and serum tubes were centrifuged at 1650 G for 15 min. Subsequently, plasma and serum were stored at −80 °C (plasma) and −20 °C (serum), respectively, until further analyses. Serum mineral contents were analysed as described for feeding materials. For determining the OC-concentrations in the plasma samples of the stallions, a commercially available enzyme-linked immunosorbent assay (ELISA) was used (N-MID^®^ Osteocalcin Enzyme-Linked Immunosorbent Assay, IDS Immunodiagnostic Systems GmbH, Frankfurt am Main, Germany), whereas CTX-I was analysed by an enzyme-linked immunosorbent assay (Serum CrossLaps^®^ ELISA, IDS Immunodiagnostic Systems GmbH, Frankfurt am Main, Germany). OC and CTX-I were analysed in blood samples of all 30 stallions at the beginning of the trial (week 0), before the beginning of phase 2 (week 10) and at the end of the trial (week 20). Analysis of OC and CTX-I was performed at the Institute of Animal Nutrition, Nutrition Diseases and Dietetics, Faculty of Veterinary Medicine, Leipzig University, Leipzig, Germany. All analyses were performed in duplicate.

### 2.5. Statistical Analysis

Statistical analyses were performed by using SAS Enterprise Guide 5.1 software (SAS Institute Inc., Cary, NC, USA). Residuals were tested for normal distribution. To test for significant differences between groups, either the Wilcoxon test or t-test for independent samples was used. In case neither the original data nor the decadic logarithm were normally distributed, the Wilcoxon test was applied. If the normal distribution was given, a t-test was used. To statistically confirm a time effect on the mineral concentrations in the serum of the stallions, either a repeated-measures analysis of variance (GLM) or the signed-rank test was used. While a repeated-measures analysis of variance was applied for normally distributed values, the signed-rank test was selected if the values were not normally distributed. Differences with a significant level of *p* < 0.05 were taken to be statistically significant.

## 3. Results

### 3.1. Health Status and Body Weight Development

Due to colic, one animal died and was therefore not included in the evaluation. Another animal was excluded from the study as it was sold. The remaining 30 stallions showed no clinical signs of disease during the experimental period. The bodyweight of the horses did not differ between the groups throughout the trial. Figure S1 graphically shows the body weight development for both groups.

### 3.2. Serum Macro Element Concentrations

Table 5 shows calcium and phosphorus concentrations in the serum of Ca-High and Ca-Moderate throughout the trial. With reduced calcium intake (Ca-Moderate), a 3.3% lower calcium value (*p* = 0.0006) and an approximately 26% higher phosphorus concentration (*p* = 0.0002) were observed in the serum of the stallions at the end of the trial (experimental week 20) compared to Ca-High. When comparing calcium serum levels of Ca-Moderate at week 0 and week 20, there was a significant decrease of 5.6%, whereas in Ca-High calcium concentrations did not differ between week 0 and week 20. Concentrations of phosphorus were higher in Ca-Moderate than in Ca-High, which was significant at week 0 (*p* = 0.0467) and week 20 (*p* = 0.0002). Phosphorus levels significantly decreased in Ca-High during the trial, whereas in Ca-Moderate concentrations at week 20 did not differ from week 0.

In terms of magnesium (Table 6), there were no significant differences in serum concentration, except for measurement at week 4 (*p* = 0.0187). Additionally, in Ca-Moderate, magnesium concentrations were lower at the end of the trial than at the beginning.

In general, the sodium concentrations (Table 6) in the serum had a relatively low level. Although all animals in both groups had a salt lick stone at their free disposal, significantly lower values were observed in Ca-Moderate than in Ca-High at week 14 (*p* < 0.0001). At the end of the experiment, however, significantly higher sodium concentrations in the serum were determined in Ca-Moderate compared to Ca-High (*p* < 0.0001).

As concentrations of potassium and chloride were not normally distributed, values in Table 7 are given as medians. The serum potassium concentrations did not differ between Ca-High and Ca-Moderate throughout the trial.

The chloride concentrations varied in the course of the trial in both groups. In Ca-Moderate, slightly but significantly lower chloride values were observed at the end of the trial compared to Ca-High (*p* = 0.0185). During the trial period, the chloride concentrations in the serum of both groups increased significantly, while the potassium concentrations in the serum did not differ significantly between the start and end of the study.

### 3.3. Serum Trace Element Concentrations

**Figure 2:**
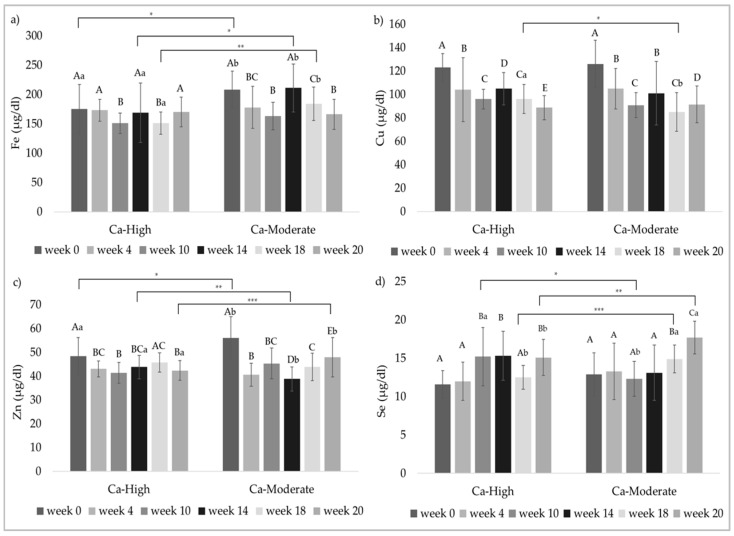
Development of the trace element concentrations in the serum of the stallions (mean ± SD, given in µg/dL). (**a**) Fe concentrations, reference values: 70–200 µg/dL; (**b**) Cu concentrations, reference value: 50–250 µg/dL; (**c**) Zn concentrations, reference value: 50–150 µg/dL; (**d**) Se concentrations, reference value: 3.95–25.3 µg/dL [14]. ^A, B, C^ Different capital letters indicate significant differences between the different measurement time points within a group. Means with common superscripts are not significantly different. ^a, b^ Different lower case letters indicate significant differences between the groups. Means with common superscripts are not significantly different. Asterisks indicate the level of significance. * *p* < 0.05; ** *p* < 0.001; *** *p* < 0.0001.

In the following, concentrations of trace elements in the serum of the stallions are graphically shown in Figure 2. Mean values, standard deviations as well as minimum and maximum values, respectively, can be found in the supplementary material Table S5.

In the course of the trial, increasing and decreasing iron concentrations in the serum were observed within both feeding groups. At week 0 (*p* = 0.0212), week 14 (*p* = 0.0230) and week 18 (*p* = 0.0010), respectively, significantly higher iron concentrations were observed in Ca-Moderate in comparison to Ca-High, which, however, could not be confirmed at the end of the trial (*p* = 0.6970).

The copper concentrations did not differ between Ca-High and Ca-Moderate except for measurement at week 18 (*p* = 0.0457). At the beginning of the study, the copper concentrations were in the range of 125 µg/dL but significantly decreased until week 20 when concentrations in both groups were in the range of 90 µg/dL.

Similar to the copper concentrations, the highest zinc values were measured at the beginning of the study. While serum zinc varied around 52.5 µg/dL at the first measurement time, the mean concentrations in the following measurements were consistently below the reference value of 50 µg/dL. The comparison of the two feeding groups did not show a clear tendency. In week 0 and week 20, the values in Ca-High were significantly lower (*p* = 0.0172 and *p* = 0.0002, respectively), whereas in week 14 they were significantly higher (*p* = 0.0098) than in Ca-Moderate.

In terms of selenium concentrations, values at week 0 varied between 7.96 and 18.6 µg/dL and did not differ between the groups. In contrast to the previously listed trace elements, the selenium concentrations increased towards the end of the study and were significantly higher than at the beginning in both groups. Furthermore, selenium concentrations in Ca-Moderate were significantly higher than in Ca-High at week 18 (*p* = 0.0006) and week 20 (*p* = 0.0038).

### 3.4. Bone Marker

**Figure 3:**
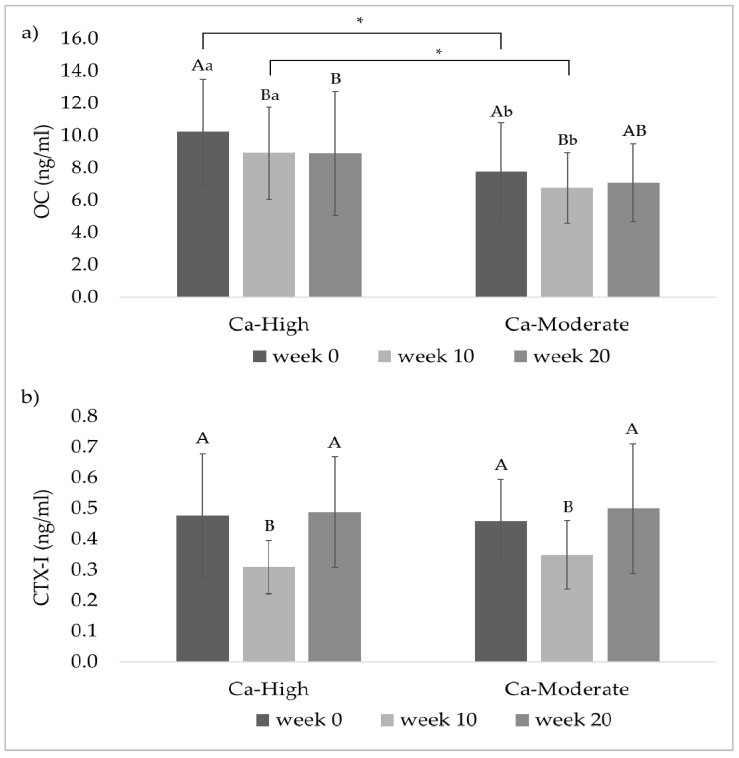
Concentrations of bone markers in the blood samples of the stallions (mean ± SD, given in ng/mL). (**a**) Osteocalcin (OC); (**b**) C-terminal Telopeptide (CTX-I). ^A, B, C^ Different capital letters indicate significance between the different measurement time points within a group. Means with common superscripts are not significantly different. ^a, b^ Different lower case letters indicate significant differences between the groups. Means with common superscripts are not significantly different. Asterisks indicate the level of significance. * *p* < 0.05.

Plasma concentrations of bone markers (OC and CTX-I) are graphically shown in Figure 3. The baseline OC concentration measured at week 0 was significantly higher in Ca-High than in Ca-Moderate (Ca-High: 10.2 ^a^ ± 3.26; Ca-Moderate: 7.75 ^b^ ± 3.05 ng/mL; *p* = 0.044). This difference was still present before the feed changed in week 10 (Ca-High: 8.90 ^a^ ± 2.84; Ca-Moderate: 6.76 ^b^ ± 2.17 ng/mL; *p* = 0.0491), whereas at the end of the trial, the groups did not differ significantly (Ca-High: 8.90 ^a^ ± 3.84; Ca-Moderate: 7.08 ^a^ ± 2.40; *p* = 0.1489). In both groups, the OC concentrations sank from week 0 to week 10 (Ca-High: *p* = 0.0093; Ca-Moderate: *p* = 0.0237), but concentrations at week 20 did not differ from those at week 10 (Ca-High: *p* = 0.3996; Ca-Moderate: *p* = 0.2713).

CTX-I concentrations were 0.476 ± 0.20 ng/mL (Ca-High) and 0.457 ± 0.14 ng/dL (Ca-Moderate), respectively, at week 0. In both groups, the concentrations decreased significantly (Ca-High: *p* = 0.0004; Ca-Moderate: *p* = 0.0027) within the first 10 weeks (Ca-High: 0.308 ± 0.09; Ca-Moderate: 0.348 ± 0.11 ng/mL) and then increased significantly (Ca-High: *p* = 0.0006; Ca-Moderate: *p* = 0.0004) to the level of the initial values at week 20 (Ca-High: 0.487 ± 0.18; Ca-Moderate: 0.499 ± 0.21 ng/mL). However, concentrations were not significantly different at any time point between the groups.

## 4. Discussion

As it is common practice to use mineral supplements in horse feeding, the aim of this study was to investigate the effects of different dietary calcium levels in young warmblood stallions under practical conditions. The investigations focused on whether reducing calcium supply offers an advantage over conventional feeding regimes in terms of trace element utilisation and, furthermore, whether bone metabolism might be affected due to either dietary treatment. Conventional feeding regimes can be understood as roughage (and oat) based rations, which are supplemented with calcium-rich complementary feeds in amounts of about 1 kg per day and animal. In the present study, this feeding regime was represented by the ration of Ca-High during phase 2. In phase 1, the supplementation of (macro) and trace elements as well as vitamins was carried out on the basis of mineral feeds which require amounts of about 0.1 kg per day per animal. During both phases, calcium supply in group Ca-Moderate was reduced in comparison to Ca-High. In general, mineral feeds are particularly suitable for horses in maintenance, as their energy requirement is covered (or even exceeded) by ad libitum feeding of hay [16], but can also be fed as a supplement to hay and concentrates in the case of increased energy requirements. However, complementary feeds replace part of the concentrates and supply horses not only with minerals and vitamins but also with a certain amount of energy and protein. The resulting larger dosage offers a degree of protection against a (potentially dangerous) over-supply of trace elements and vitamins, as dosage inaccuracies are relatively less significant.

Considering mineral intake from 8 kg hay as fed in the present study displayed and compared to requirements according to GfE [1] in Table S4, the horses in the present study were already sufficiently supplied with macro minerals (except for sodium) by the hay. In addition, an uptake of straw (approx. 2 kg DM per animal and day) from the bedding material can also be assumed. This resulted in an additional supply of 4.4–4.8 g calcium, 3.0–3.7 g phosphorus, 18.8–20.2 g potassium, and 4.0–8.2 g chloride considering the chemical composition of the straw used in this study (Table S2), whereas an additional supply of magnesium and sodium was negligible. With regard to trace elements, this study revealed clear deficits in the supply of copper, selenium and zinc, when hay (and straw) was the only source for minerals. In the literature, the macro element contents in hay are quantified similarly high. An accredited service laboratory in Germany (Landwirtschaftliche Untersuchungs- und Forschungsanstalt, LUFA Nord-West, Oldenburg, Germany) found in hay samples from 2014 to 2019 mean concentrations (and ranges of variations) of 4.7–4.9 g (1.6–19.4 g) Ca/kg DM, 2.2–2.6 g (0.6–4.9 g) P/kg DM, 0.2–1.4 g (< 0.2–11.1 g) Na/kg DM, 1.7–1.9 g (0.6–5.6 g) Mg/kg DM and 17.7–19.4 g (4.2–37.8 g) K/kg DM, respectively [2]. Concentrations of trace elements were also comparable to those of the present study with 4.9–5.9 mg (2.1–11.2 mg) Cu/kg DM and 25–32 (10–111) mg Zn/kg DM [2], so it can be assumed that the mineral concentrations in hay measured in this study are in line with the average and do not represent an outlier.

Warmblood horses comprise a large part of the horse population in Europe. However, there is hardly any data on the effects of different calcium contents in the diet of warmblood horses on mineral household and bone metabolism. Within the population of warmblood horses, the animals from the present study represent a homogenous group as only stallions of the same age belonging to one breed were used. While in mares, there are differences in the mineral status in dependence of reproductive status, mineral metabolism in stallions is subject to less fluctuations due to reproduction [19].

Since blood calcium levels are tightly regulated by endocrine mechanisms, blood calcium levels in the present study did barely differ in dependence on dietary calcium supply and all measured values were within the reference of 9.6–13.6 mg/dL [10,16]. When comparing the serum phosphorus concentrations on the dependence of high and moderate calcium supply, it was noticeable that in Ca-Moderate, the phosphorus levels were numerically higher during the trial and significantly higher at the end of the trial in spite of slightly lower dietary phosphorus intake. The same observation could already be made in a balance study with adult ponies, where the effects of low compared to high calcium contents in the diet were likewise investigated [20]. It can be assumed that calcium and phosphorus form complexes in the intestinal content, which reduces the bioavailability of phosphorus, especially in the presence of high dietary calcium levels [21]. Furthermore, calcium carbonate, which was used in CF_1_, works as a buffer and can increase intestinal pH. As phosphorus absorption is decreased with increasing intestinal pH, this might have further reduced the phosphorus availability in Ca-High [21]. Nevertheless, all phosphorus serum levels were within the reference value [16].

The labour of the horses (30 min/day), which according to Coenen and Vervuert [16] can be classified as “light”, results in an additional requirement for macro elements, which is mainly due to electrolyte losses via sweat. However, the additional requirement for calcium, phosphorus and magnesium is already covered by the increased feed intake necessary to meet the energy requirement [1]. The most abundant minerals in horse sweat are sodium (3.1 g/L), chloride (5.5 g/L), and potassium (1.6 g/L) [22]. Since the sweat production of the horses was not monitored, it was not possible to quantify the amount of losses through sweat. Next to sweat production during work, basal sweat production should also be taken into account. According to Coenen and Vervuert [16], basal sweat production is about 0.04l/kg BW ^0.75^ resulting in 4.5l of sweat per day and animal for the stallions in the present study. Basal sweat losses already occur within the thermoneutral zone (5–25 °C) of horses and increase at temperatures above 25 °C [23]. In terms of sodium and chloride, intake through the salt-lick-stones is also an unknown variable, so that the sodium contents in the diet given in Table 2 do not represent the total intake. However, the measured sodium concentrations were frequently below the reference range of 303–336 mg/dL [16] in both groups, even though daily sodium intake exceeded recommendations of the GfE [1]. These results support the assumption by Coenen and Vervuert [16] that voluntary sodium intake via a salt-lick-stone might be not sufficient to meet the performance requirement for sodium that can reach amounts of 60 g sodium per day in hard-working warmblood horses (600 kg BW). For horses that sweat regularly, offering loose salt together with the concentrates should therefore be considered [16]. Furthermore, in line with the low sodium serum levels measured in this study and against the background of basal sweat production according to Coenen and Vervuert [16], the recommendation of the GfE on the maintenance requirement of sodium of 3.4 g per day for a warmblood horse [1] could be reconsidered. The statements made for sodium also apply in principle to chloride. Serum chloride was several times below the reference of 351–386 mg/dL [16], although chloride requirements were already fulfilled due to hay [1]. Potassium concentrations were independent of the treatment mostly within the reference range of 10.9–18.8 mg/dL [16]. All 13 exceptions were below 10.9 mg/dL. Similar to sodium and chloride serum levels, losses via sweat could be the cause. However, studies on the influence of sweating on the potassium concentrations in the blood provide contradictory results. While Soliman and Nadim [24] found a decrease in serum potassium after exercise, Assenza et al. [25] reported increasing potassium levels in the serum after three days of intensive training which was assumed to result due to losses from muscle fibres.

To evaluate the dietary supply of macro elements, blood tests provide a rapid and easy way of assessment. However, as already mentioned for calcium levels, limitations must be made with regard to the reliability of the results due to endocrine regulatory mechanisms [26]. Furthermore, variations can occur as a consequence of circadian, prandial or genetic variations [26], which, however, were excluded as best as possible in the present study.

In phase 1, the copper supply of Ca-High was 87.7 mg/d which is about 30% below the recommendations of the GfE from 2014 [1]. This was caused mainly by the very low copper content of the hay (3.03 mg/kg DM) in the first phase of the trial, while in Ca-Moderate the copper requirement was met due to slightly higher copper concentrations in MF_2_ than in MF_1_. In phase 2, the copper supply for both groups was in the range of requirement. However, all serum copper levels were within the reference range of 50–250 µg/dL [16]. Nevertheless, it should be considered that copper storage in the liver can be depleted in terms of insufficient supply [27]. A 14-day feeding trial in mares revealed that mane hair might be a useful tool to evaluate dietary copper supply and therefore was recommended by the authors to use instead of blood sampling [28]. The zinc supply of Ca-High in phase 1 met the requirement (525 mg/d) while it was in phase 2 and in Ca-Moderate during both phases slightly above the requirement [1]. However, despite the slightly lower zinc supply in Ca-High, the high calcium excess had no effect on zinc levels in the serum in comparison to Ca-Moderate. In both groups, zinc levels were partly too low [16]. Among the complementary feeds used, zinc was added in the form of inorganic zinc (zinc oxide and zinc sulphate). When evaluating the zinc supply, the zinc source should be taken into account, as inorganic zinc sources are associated with a lower bioavailability than organic zinc compounds [29]. In addition, there is an interaction between dietary zinc and iron. It has been shown for several animal species that high levels of iron (as in the rations used in this study, presented in Table 2) can impair zinc utilisation [30]. The high iron concentrations in the serum, which partly exceeded the reference level according to Coenen and Vervuert [16] of 70–200 mg/dL, could also be explained by the high iron contents in the diet which exceeded the requirements [1] by 280%. However, when using the reference range of 70–300 mg/dL according to Geor et al. [31], all values were within the norm. Considering the entirety of measured trace element concentrations in serum, the hypothesis of an influence of high calcium excess on serum trace element concentrations cannot be confirmed. Therefore, it can be assumed that serum levels of trace elements are subject to a variety of influences and do not always reflect the level of supply.

As calcium together with phosphorus is the most abundant mineral in the bone, playing an important role for bone formation especially during growth, a further focus of the study was bone metabolism. Osteocalcin (OC), as well as C-terminal telopeptide (CTX-I), are reliable markers in the blood to reflect bone formation (OC) and degradation (CTX-I) processes [12,13]. In the present study, marker concentrations were determined at the beginning of the trial, before the change of MF_1_ to CF_1_ and MF_2_ to CF_2_, respectively, in week 10, and at the end of the trial. In terms of OC, concentrations were already significantly higher in Ca-High at the first measurement (Ca-High: 10.2 ^a^ ± 3.26; Ca-Moderate: 7.75 ^b^ ± 3.05 ng/mL). As this finding cannot be attributed to the dietary treatment, it was checked whether other factors could have caused the difference. According to Lepage et al. [12], OC concentrations fall with age. However, the mean days of the age of both groups were identical (Ca-High: 965 ± 19.1; Ca-Moderate: 964 ± 28.9 d). Bodyweight cannot be accountable for the difference either, as this did not differ between the groups during the entire experimental period. Overall, the values in this study were comparatively low. In Lusitano horses of different age groups, OC concentrations in blood were 35.4 ± 5.0 ng/mL (24 months old) and 26.2 ± 5.7 ng/mL (36 months old), respectively [32]. Similar results were obtained by Jackson et al. [33] who found OC concentrations of 37.0 ng/mL in two-year-old and 29.9 ng/mL in three-year-old thoroughbreds. The varying levels could be due to the use of different testing methods. While OC concentrations in the present study were analysed by means of an ELISA (IDS Immunodiagnostic Systems GmbH, Frankfurt am Main, Germany), OC levels in the study by Fradinho et al. [32] and Jackson et al. [33] were determined by means of a competitive immunoassay (QUIDEL Corporation, San Diego, CA, USA). However, our results are in agreement with Porr et al. [34], who also could not demonstrate differences in serum OC concentrations due to either low- or high-calcium diets in adult Arabian horses. With respect to CTX-I concentrations, there were no differences between the groups throughout the trial. Concentrations in both groups were significantly lower at week 10 than at the beginning of the trial but reached the initial level again at the end of the trial. Fluctuations in CTX-I concentrations were demonstrated in dependence on workload and movement [35]. Before the start of the trial, horses were kept in groups in playpens. After horses were brought to the stallion station where they were kept in individual boxes, the duration of movement was reduced. Therefore, CTX-I concentrations might have decreased. However, during the second half of the trial intensity of movement was increased as the horses have been broken in. This might explain the rise in CTX-I concentrations. Nevertheless, CTX-I concentrations in this study are consistent with values in two-year-old (0.46 ng/mL) and three-year-old (0.38 ng/mL) thoroughbreds [33].

## 5. Conclusions

In summary, the hypothesis of a higher efficiency in the utilisation of trace elements by reducing the intake of calcium could not be confirmed in this feeding trial. Furthermore, different dietary levels of calcium did not influence bone metabolism in growing warmblood horses. However, results indicate that forage-based rations for horses do not necessarily have to be supplemented with calcium but with trace elements.

## Figures and Tables

**Table 1:** Feed supply per animal and day (kg FM ^1^, as fed).

Experimental Phase	Feed Item	Ca-High	Ca-Moderate
Phase 1	Hay	8.00	8.00
	Oat	2.25	2.25
	Concentrates	0.80	0.80
	Soybean meal	0.10	0.10
	Plant oil	0.05	0.05
	MF_1_	0.12	---
	MF_2_	---	0.12
Phase 2	Hay	8.00	8.00
	Oat	2.25 ↑^2^	2.25 ↑^2^
	Plant oil	0.05	0.05
	CF_1_	1.00	---
	CF_2_	---	1.00

^1^ FM, fresh matter. ^2^ Minimal supply 2.25 kg oats per day, optionally more when horses lost weight.

**Table 2:** Chemical composition of mineral feeds (MF_1_, MF_2_) and complementary feeds (CF_1_, CF_2_).

Item	Unit	Phase 1		Phase 2	
		MF_1_	MF_2_	CF_1_	CF_2_
Crude ash	g/kg DM ^1^	802	80.0	91.4	64.6
Crude protein	g/kg DM	17.7	125	242	239
Ether extract	g/kg DM	5.17	39.8	68.3	66.9
Crude fibre	g/kg DM	17.6	157	77.0	78.2
Nitrogen-free extract (NfE) ^2^	g/kg DM	158	598	521	551
Calcium	g/kg DM	204	11.2	18.4	7.96
Phosphorus	g/kg DM	42.0	4.81	8.04	5.78
Magnesium	g/kg DM	23.7	3.79	2.91	2.71
Sodium	g/kg DM	47.2	6.29	2.23	2.56
Potassium	g/kg DM	1.66	6.02	9.77	10.0
Chloride	g/kg DM	80.4	4.27	3.58	4.04
Sulphur	g/kg DM	1.30	5.59	3.01	3.16
Iron	mg/kg DM	1817	1106	1036	975
Copper	mg/kg DM	357	809	91.2	95.7
Zinc	mg/kg DM	1269	2518	378	435
Manganese	mg/kg DM	831	1288	289	311
Selenium	mg/kg DM	14.7	20.1	1.62	2.06

^1^ DM, dry matter. ^2^ Nitrogen-free extract (NfE) = dry matter—(ash + crude protein + ether extract + crude fibre). Sources of added macro elements: MF_1_: Ca as Ca(H_2_PO_4_)_2_ **•** H_2_O, Mg as MgO and Mg_4_Si_6_O_15_(OH)_2_ **•** 6 H_2_O (replaced by apple pomace and wheat semolina bran in MF_2_). CF_1_: Ca as Ca(H_2_PO_4_)_2_ **•** H_2_O and CaCO_3_ (replaced by maize in CF_4_). Sources of added trace elements: Fe as FeCO_3_, Cu as CuSO_4_ **•** 5 H_2_O, Zn as ZnO and ZnSO_4_ **•** H_2_O, Mn as MnO, Se as Na_2_SeO_3_.

**Table 3:** Mineral supply ^1^ per stallion per day in the two experimental phases, categorised by group compared to requirements ^2^.

Mineral	Unit	Requirement ^2^	Phase 1		Phase 2	
			Ca-High	Ca-Moderate	Ca-High	Ca-Moderate
Calcium	g/kg DM	25.8	65.4	42.7	49.4	39.8
Phosphorus	g/kg DM	16.6	36.8	32.4	37.3	35.1
Magnesium	g/kg DM	6.50	18.5	16.2	17.9	17.7
Sodium	g/kg DM	3.40	17.3	12.5	8.32	8.59
Potassium	g/kg DM	16.8	124	124	155	155
Chloride	g/kg DM	2.0	81.6	72.6	71.9	72.3
Iron	mg/kg DM	520	2651	2558	2116	2050
Copper	mg/kg DM	130	87.7	133	122	125
Zinc	mg/kg DM	525	531	654	675	722
Selenium	mg/kg DM	1.75	2.22	2.66	1.61	1.99

^1^ Calculated according to ration design in Table 1; also corresponds approximately to intake, since no feed refusals were observed; Straw intake from the bedding was estimated. ^2^ Requirement according to GfE [1].

**Table 4:** Energy and crude nutrient supply ^1^ per stallion per day in the two experimental phases, categorised by group.

Item	Unit	Phase 1		Phase 2	
		Ca-High	Ca-Moderate	Ca-High	Ca-Moderate
Metabolisable energy (ME) ^2^	MJ/day	95.1	95.1	97.1	97.4
Crude ash	g/day	758	673	589	564
Crude protein	g/day	1048	1060	1109	1104
Ether extract	g/day	265	269	278	276
Crude fibre	g/day	3598	3613	3744	3744

^1^ Calculated according to ration design in Table 1; also corresponds approximately to intake, since no feed residues were observed; straw intake from the bedding was estimated. ^2^ Metabolisable energy (ME) calculated from specific nutrient content according to GfE [1].

**Table 5:** Calcium and phosphorus serum concentrations of the stallions (mean ± SD; min–max), given in mg/dL.

Week		Ca		P	
		Ca-High	Ca-Moderate	Ca-High	Ca-Moderate
0	Mean ± SD	12.4 ± 0.453	12.5 ^A^ ± 0.444	3.40 ^Aa^ ± 0.471	3.71 ^ACb^ ± 0.346
	min–max	11.7–13.1	11.6–13.1	2.36–4.06	3.21–4.59
4	Mean ± SD	12.4 ± 0.728	12.3 ^AB^ ± 0.669	3.34 ^AB^ ± 0.568	3.81 ^A^ ± 0.738
	min–max	11.4–14.7	10.7–13.0	2.27–4.28	2.55–4.90
10	Mean ± SD	12.4 ± 0.508	12.4 ^AB^ ± 0.211	3.19 ^AC^ ± 0.361	3.36 ^BC^ ± 0.279
	min–max	11.5–13.7	11.9–12.6	2.53–3.78	2.86–3.86
14	Mean ± SD	12.5 ^a^ ± 0.539	12.1 ^Bb^ ± 0.382	3.33 ^AC^ ± 0.405	3.59 ^AC^ ± 0.391
	min–max	11.3–13.6	11.5–12.8	2.52–4.26	2.90–4.45
18	Mean ± SD	12.2 ± 0.480	12.3 ^AB^ ± 0.344	3.04 ^C^ ± 0.323	3.10 ^B^ ± 0.549
	min–max	11.5–12.8	11.8–13.1	2.46–3.64	1.98–4.09
20	Mean ± SD	12.2 ^a^ ± 0.337	11.8 ^Cb^ ± 0.311	3.08 ^CBa^ ± 0.394	3.88 ^Ab^ ± 0.598
	min–max	11.8–12.8	11.3–12.6	2.35–3.67	2.63–4.90

^a, b^ Different superscript lowercase letters indicate significant differences between Ca-High and Ca-Moderate within either Ca or P concentrations. Means with common small superscripts are not significantly different. ^A, B, C^ Different superscript capital letters indicate significant differences between the different measurement times within one group. Means with common capital superscripts are not significantly different. Reference values: Ca: 9.6–13.6 mg/dL; P: 2.17–5.27 mg/dL [16].

**Table 6:** Magnesium and sodium serum concentrations of the stallions (mean ± SD, min–max), given in mg/dL.

Week		Mg		Na	
		Ca-High	Ca-Moderate	Ca-High	Ca-Moderate
0	Mean ± SD	1.89 ^AC^ ± 0.314	1.94 ^A^ ± 0.135	299 ^A^ ± 23.5	301 ^A^ ± 7.08
	min–max	1.39–2.68	1.63–2.12	266–375	289–315
4	Mean ± SD	1.85 ^ABCa^ ± 0.119	1.99 ^Ab^ ± 0.171	276 ^B^ ± 14.5	285 ^B^ ± 18.7
	min–max	1.65–2.10	1.74–2.28	252–299	259–320
10	Mean ± SD	1.76 ^B^ ± 0.112	1.79 ^B^ ± 0.105	296 ^A^ ± 10.6	292 ^BD^ ± 7.27
	min–max	1.57–1.96	1.57–2.07	280–322	283–308
14	Mean ± SD	1.94 ^C^ ± 0.136	1.92 ^AC^ ± 0.125	300 ^Aa^ ± 8.99	269 ^Cb^ ± 20.9
	min–max	1.70–2.17	1.70–2.16	289–316	237–304
18	Mean ± SD	1.82 ^AB^ ± 0.128	1.86 ^BC^ ± 0.085	297 ^A^ ± 4.57	298 ^AD^ ± 7.50
	min–max	1.59–2.03	1.73–2.01	287–305	284–309
20	Mean ± SD	1.87 ^AC^ ± 0.119	1.86 ^BC^ ± 0.102	279 ^Ba^ ± 7.79	294 ^ADb^ ± 6.10
	min–max	1.69–2.07	1.66–2.03	268–294	286–311

^a, b^ Different superscript lowercase letters indicate significant differences between Ca-High and Ca-Moderate within either Mg or Na concentrations. Means with common small superscripts are not significantly different. ^A, B, C, D^ Different superscript capital letters indicate significant differences between the different measurement times within a group. Means with common capital superscripts are not significantly different. Reference values: Mg: 1.22–2.92 mg/dL; Na: 303–336 mg/dL [16].

**Table 7:** Potassium and chloride serum concentrations of the stallions (median, min–max), given in mg/dL.

Week		K		Cl	
		Ca-High	Ca-Moderate	Ca-High	Ca-Moderate
0	Median	15.6 ^AB^	14.6	343 ^AC^	343 ^AC^
	min–max	10.5–17.0	10.0–17.3	290–361	302–359
4	Median	14.9 ^A^	15.6	363 ^Ba^	343 ^ACb^
	min–max	8.44–17.6	10.6–17.9	354–367	333–361
10	Median	16.1 ^B^	15.5	350 ^AD^	346 ^A^
	min–max	11.8–18.3	12.0–17.1	335–363	335–362
14	Median	15.4 ^AB^	15.3	338 ^C^	341 ^C^
	min–max	12.0–17-1	12.0–17.6	308–361	310–350
18	Median	14.8 ^A^	15.2	343 ^AC^	341 ^AC^
	min–max	8.28–16.8	8.60–18.1	331–361	331–356
20	Median	15.4 ^AB^	15.6	360 ^BDa^	351 ^Bb^
	min–max	9.80–17.4	11.1–17.6	346–397	336–384

^a, b^ Different superscript lowercase letters indicate significant differences between Ca-High and Ca-Moderate within either Mg or Na concentrations. Means with common small superscripts are not significantly different. ^A, B, C, D^ Different superscript capital letters indicate significant differences between the different measurement times within a group. Means with common capital superscripts are not significantly different. Reference values: K: 10.9–18.8 mg/dL; Cl: 351–386 mg/dL [16].

## Data Availability

The data presented in this study are available in this manuscript and the supplementary material.

## Supplementary Materials

The following are available online at https://www.mdpi.com/article/10.3390/ani11082439/s1, Table S1: Initial and final bodyweight (BW) as well as initial age of the stallions. Table S2: Chemical composition of the concentrates, oats, soybean meal, and straw, Table S3: Chemical composition of the hay used in phase 1 and phase 2. Table S4: Requirements of 600 kg warmblood horses in the age of 25 to 36 months and supply of minerals from 8 kg hay as fed in phase 1 and phase 2. Table S5: Concentrations of trace elements in the serum samples of the stallions (mean ± SD, min–max, µg/dL). Figure S1: Body weight development of Ca-High (*n* = 14) and Ca-Moderate (*n* = 16) during the experimental period.
