# Supplementary material for: Investigations on the Effects of Different Calcium Supply Exceeding the Requirements on Mineral Serum Concentrations and Bone Metabolism in Young Warmblood Stallions
Source: Animals (Basel). 2021 Aug 19;11(8):2439. doi: 10.3390/ani11082439 (PMC8388694; doi:10.3390/ani11082439)
Supplement: Supplementary file 1 [file animals-11-02439-s001.zip › animals-1301388-supplementary.pdf]

Supplementary Materials

Table S1. Initial and final Bodyweight (BW) as well as initial age of the stallions.

Horse ID	BW (kg) week 0	BW (kg) week 20	Age (d) week 0
	**Group 1**		
1	470	500	943
2	560	560	941
3	580	590	979
4	570	580	988
5	500	540	954
6	550	580	924
7	480	500	956
8	530	540	970
9	540	550	976
10	540	560	993
11	550	570	960
12	530	540	971
13	530	550	953
14	530	550	969
31 *	500		
32 **	530		
	**Group 2**		
15	560	580	941
16	490	520	932
17	530	540	951
18	560	580	967
19	530	540	941
20	550	570	979
21	530	530	976
22	540	550	946
23	560	570	998
24	510	520	998
25	550	570	931
26	610	580	1000
27	480	530	990
28	500	550	1011
29	570	570	908
30	540	550	958

* Died as a result of colic. ** Sold.

Table S2. Chemical composition of the concentrates, oats, soybean meal (SBM) and straw.

Item	Unit	Concentrates	Oats	SBM	Straw phase 1	Straw phase 2
Crude ash	g/kg DM[1]	79.9	875	71.6	76.1	41.5
Crude protein	g/kg DM	131	21.6	517	51.0	32.3
Ether extract	g/kg DM	24.8	115	19.3	5.86	8.28
Crude fibre	g/kg DM	127	46.9	67.0	449	493
NDF[2]	g/kg DM	344	104	23.9	830	731
Calcium	g/kg DM	14.7	372	3.75	2.22	2.42
Phosphorus	g/kg DM	4.53	0.993	7.05	1.92	1.48
Magnesium	g/kg DM	1.85	3.72	3.30	0.454	0.399
Sodium	g/kg DM	2.20	1.20	0.34	0.0436	0.0827
Potassium	g/kg DM	12.6	0.0299	24.0	10.1	14.4
Chloride	g/kg DM	4.37	0.809	1.02	4.11	2.02
Iron	mg/kg DM	409	92.9	307	69.8	52.2
Copper	mg/kg DM	19.1	3.75	20.5	1.61	2.57
Zinc	mg/kg DM	131	35.6	54.5	6.02	17.9
Manganese	mg/kg DM	108	42.7	42.0	9.36	18.9
Selenium	mg/kg DM	0.435	0.010	0.239	0.033	0.010

[1] DM, dry matter. [2] NDF, neutral detergent fibre.

Table S3. Chemical composition of the hay used in phase 1 and phase 2.

Item	Unit	Phase 1	Phase 2
Crude ash	g/kg DM[1]	55.5	51.4
Crude protein	g/kg DM	86.4	82.5
Ether extract	g/kg DM	13.1	14.6
Crude fibre	g/kg DM	322	329
NDF[2]	g/kg DM	705	707
Calcium	g/kg DM	3.43	3.52
Phosphorus	g/kg DM	2.41	2.70
Magnesium	g/kg DM	1.57	1.68
Sodium	g/kg DM	1.43	0.845
Potassium	g/kg DM	11.9	14.8
Chloride	g/kg DM	8.24	8.75
Sulphur	g/kg DM	1.91	1.65
Iron	mg/kg DM	244	121
Copper	mg/kg DM	3.02	3.51
Zinc	mg/kg DM	29.4	30.6
Manganese	mg/kg DM	193	167
Selenium	mg/kg DM	0.014	0.012

[1] DM, dry matter. [2] NDF, neutral detergent fibre.

Table S4. Requirements of 600 kg warmblood horses[1] in the age of 25 to 36 months and supply of minerals from 8 kg hay as fed in phase 1 and phase 2.

Mineral	Unit	Requirement	Hay phase 1	Hay phase 2
Ca	g/d	25.8	24.2	25.1
P	g/d	16.6	17.0	19.3
Mg	g/d	6.50	11.1	12.0
Na	g/d	3.40	10.1	6.02
K	g/d	16.8	83.9	106
Cl	g/d	2.0	58.1	62.4
Fe	mg/d	520	1722	863
Zn	mg/d	525	208	218
Cu	mg/d	130	21.3	25.0
Mn	mg/d	525	1363	1191
Se	mg/d	1.75	0.099	0.086

[1] Requirement according to GfE 2014 [1].

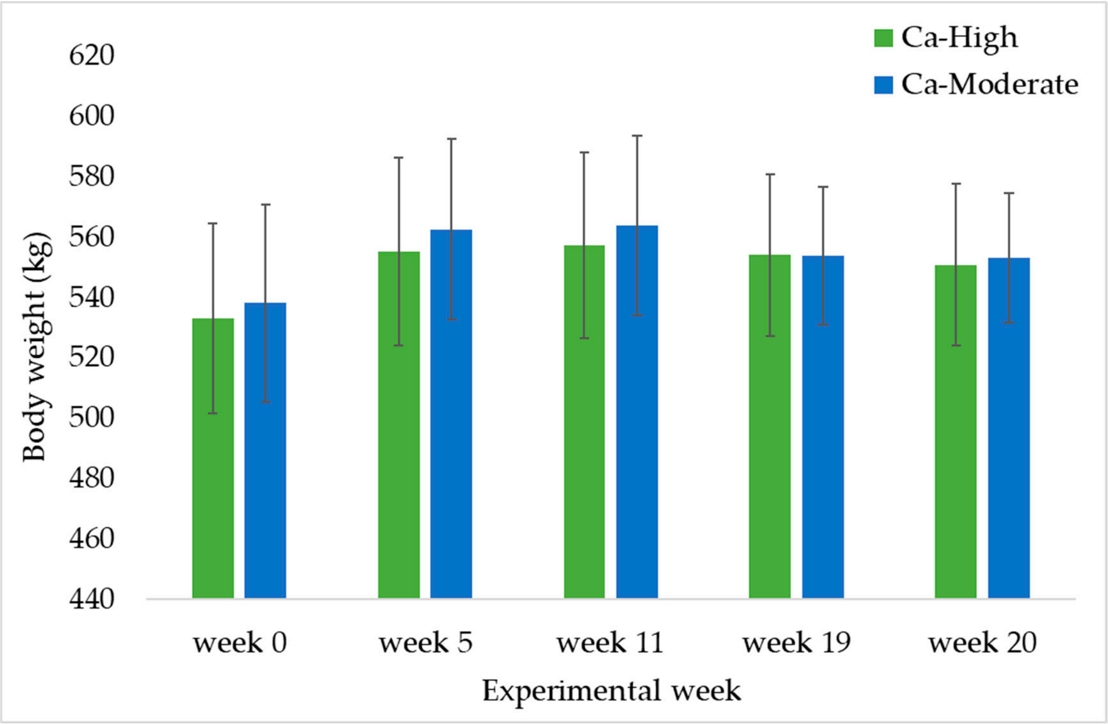

Figure S1. Body weight development of Ca-High (n = 14) and Ca-Moderate (n = 16) during the experimental period.

Table S5. Concentrations of trace elements in the serum samples of the stallions (mean ± SD, min – max, μg/dl).

Week		Fe		Cu		Zn		Se	
		Ca-High	Ca-Moderate	Ca-High	Ca-Moderate	Ca-High	Ca-Moderate	Ca-High	Ca-Moderate
0	Mean ± SD	175 ± 42.1	208 ± 31.7	123 ± 11.7	126 ± 20.2	48.4 ± 7.79	56.1 ± 8.81	11.6 ± 1.80	12.9 ± 2.82
	min - max	117-252	152-271	107-142	101-177	32.7-61.4	43.1-76.4	7.96-14.1	8.84-18.6
4	Mean ± SD	173 ± 18.5	178 ± 36.0	104 ± 27.2	105 ± 17.3	43 ± 3.43	40,5 ± 4.84	12.0 ± 2.48	13.3 ± 3.68
	min - max	152-207	116-240	15-125	72.4-133	36.9-46.9	33.7-51.4	5.69-16.4	7.35-20.6
10	Mean ± SD	151 ± 17.4	163 ± 23.3	96.1 ± 8.40	90.8 ± 10.6	41.4 ± 4.37	45.3 ± 6.49	15.2 ± 3.79	12.3 ± 2.28
	min - max	123-182	136-217	82.1-110	76.4-107	34.3-48.4	35.4-56.9	9.46-21.4	8.59-17.9
14	Mean ± SD	169 ± 50.7	211 ± 40.9	105 ± 13.8	101 ± 27.1	43.8 ± 4.90	38.8 ± 5.05	15.3 ± 3.20	13.1 ± 3.60
	min - max	18.0-226	136-280	85.1-134	19.2-135	35.7-51.9	27-45.7	10.9-21.2	6.39-19.8
18	Mean ± SD	151 ± 19.0	184 ± 28.6	96.2 ± 12.3	84.9 ± 16.5	45.7 ± 4.10	43.8 ± 5.78	12.5 ± 1.54	14.9 ± 1.83
	min - max	125-193	146-232	71.3-110	59.6-108	39.1-53.3	32.0-56.9	10.3-15.4	11.3-17.6
20	Mean ± SD	170 ± 25.3	166 ± 25.9	88.7 ± 10.2	91.5 ± 15.6	42.3 ± 4.14	47.9 ± 8.30	15.1 ± 2.35	17.7 ± 2.13
	min - max	134-213	123-206	69.6-105	63.4-116	36.0-48.6	19.9-56.6	11.6-20.3	13.7-20.7
